# The Voluntary Hospital: The Discussion

**Published:** 1914-06-27

**Authors:** 


### THE DISCUSSION.

Mr. Councillor W. J. Sanderson, the Vice-Chairman of the house committee of the Newcastle Infirmary, and an ex-Lord Mayor of the city, being in the chair.

### Mr. W. G. Carnt, Manchester, said that as the representative of the Manchester Royal Infirmary, which had been referred to in Dr. Hume's paper, he should like to thank Dr. Hume for his excellent contribution. He should like to say that the figures quoted by Dr. Hume were absolutely accurate, but it was necessary to go back a few years to arrive at a correct conclusion from a study of those figures, as instead of those figures being a condemnation of the voluntary system, they were an indication that the voluntary system was very much alive and that it was not a failure. He only wished to go back six or seven years. In 1906 the income, that was the ordinary income, of the Manchester Royal Infirmary was £22,000 per annum, and the expenditure about £20,000 per annum. The hospital was in the happy position of being able to save £2,000 from the ordinary income over and besides its legacies. That had been the case for many years. In 1908 the infirmary removed from an old institution like Newcastle to a new one, and from 292 beds, with 280 beds in actual use, it suddenly became a hospital of 493 beds, with all of them in use, which this year had been increased to 553 beds. The cost of the erection of that hospital was half a million of money, £400,000 being raised from the sale of the old site, and over £100,000 being raised by voluntary contributions. It had enabled the number of patients treated to rise from 5,000 to upwards of 8,000. When by one jump in one year the number of patients increased to such an extent it could not be reasonably expected that the income would in one year increase equally to pay the whole cost of the work. It necessarily had to be a question of time, and he wished to give them figures to show that the voluntary system, coupled with the contributions for maintenance of patients, which were also of a voluntary character, caused no hardship to be inflicted on any patient. The patients were asked if they could contribute, and what they would like to contribute, and any offer they made was accepted. He wished to point out that the system at Manchester had been most successful, and that the voluntary system in Manchester, as shown by the Royal Infirmary there, was not a failure. The ordinary income in 1906 was £22,000 for the year, in 1913, in round figures, it was £35,000, so that between 1906 and 1913 the ordinary income had increased by £13,000. The expenditure on the hospital was in 1906 £20,000; it was now £35,500, an increase of £15,500. In giving figures he wished to refer to their infirmary only because the Convalescent Home was always in the predicament of having a deficiency of £2,500 a year. In 1907 they took £86,000 out of the capital of their infirmary, and in respect of the Convalescent Home they were able to wipe out an accumulated deficiency of many years. Since then they had paid from legacies a matter of £2,000 per annum to keep the Home going. They took a large sum out of the capital, and in that way they reduced their income from investments to help the Convalescent Home, and yet in spite of that their income increased from £22,000 to £35,000 a year. They opened their new infirmary in 1909, and when they went in the first year they had a deficiency of £8,000. Last year their deficiency, in spite of their increased expenditure, was only £3,000. So that really he did not think that the voluntary system was dead. Let them take Manchester, give them a couple of years, and under similar conditions to those they had had they would have no deficiency. It would be an awful pity if the voluntary system of hospitals were changed for any other system. In his opinion there was much more money contributed to hospitals to-day than there was contributed ten years ago, and he felt sure that there was still life for the voluntary hospitals. It would be a very great pity indeed if it ever became necessary for the voluntary hospitals in this country to become municipalised.

Sir Henry Burdett said that they were all indebted to Dr. Hume, the writer of the paper, for having done a great service to the voluntary hospitals and the sick of this country. There was no doubt that at the present time, so far as hospitals were concerned, they were at the beginning of a crisis. The politicians in this country were not going to have it all their own way, and that there was no Chancellor of the Exchequer alive, whatever his politics might be, who was strong enough, or ever would be strong enough, to destroy the voluntary system. That was a matter quite apart from politics. It was simply a matter of statesmanship, and statesmanship was one of those things which we sadly lacked in the present day in the United Kingdom of Great Britain and Ireland. Now, Dr. Hume had spent—he would not say long days and sleepless nights—but he must have spent a very great deal of time upon this question, for which they were eminently his debtors. Although Dr. Hume, naturally, had fallen into errors, still, he had brought to bear upon the question a clear, an impartial, and an unbiassed mind, and his paper would mark a departure, because it was the beginning of a new attitude towards hospitals on the part of the medical profession, and, he hoped, of the public at large. Dr. Hume had told them that the voluntary system was a failure. He put it in this way: He said that voluntary aid was on its trial. Well, voluntary aid had been on its trial for a great many more years than any man in that room had lived; it had been on its trial for centuries, and it had saved this country from most of the cruelties, the wrongs, and the dreadful abuses which had attached to the treatment of the sick in almost every other country in the world. That was, perhaps, a very strong statement to make, but it was the conviction of his life, based on a careful study and a careful inspection of hospitals, where science had pre-eminent consideration and where humanity had very little play. They could not and would not have State hospitals in preference to voluntary hospitals. He would sooner die—and thousands said the same thing—than live in a country where they had only State hospitals. He had talked the matter over with different scientists. State hospitals had meant that it was the professor who dominated and humanity which suffered. The one great blessedness of the voluntary system was that the first considerations were the claims and interests of the patients. If they had gone with him and been as he had in great hospitals, magnificently equipped and conducted, which contained many things some British hospitals had not, but desired, and tried to put themselves in the position of the patients, they would feel it was good we still had in this country the voluntary system. Voluntary aid was not on its trial.

### Voluntary Aid and National Insurance.

As to the National Insurance Act, the Chancellor of the Exchequer had talked about adequate medical relief, but he had not provided it. Nor had he provided anything for the speedy restoration of the breadwinner to health which the hospitals secured in the in-patient departments, the most expensive and important of all their work. They had been told that the voluntary hospitals would be swamped by the Insurance Act. But that was not the case. Those who had started the voluntary hospitals and who were responsible for them had the fear of God in them, which was a great thing. They could not in dealing with the sick give up their responsibility and stewardship. It was to the credit of our hospital managers, without a single exception, that their voluntary hospitals were in the position they were in to-day. Having told them that voluntary aid was on its trial—it was on its trial not in the sense that it had been a failure but in the sense that it had the burden of National Insurance to face, and it had already faced it nobly—the conclusions which Dr. Hume came to were to prepare the way for and ultimately to place our hospitals under a hospital authority to be approved by the county borough and county councils. This was indeed a revolutionary proposal. It was financially unsound, because the Poor-Law Commission, after a good deal of inquiry, found that it would require £20,000,000 a year to set up a system of State hospitals in this country. If Dr. Hume's proposal were to go through it would mean a loss of revenue of £5,000,000 a year, which was at present given voluntarily for the support of the hospitals which we had; the removal of the protection of the hospital patients' interests, which the voluntary system afforded; and the ultimate dissolution of the principles underlying their duty and privilege to the sick. If Dr. Hume by his scheme were to add twenty millions a year to the local rates of this country, neither he nor the authors of such a proposal, they might depend upon it, would be appointed representatives of the ratepayers.

### Dr. Hume's Scheme in Newcastle.

But how would such a scheme affect the Royal Victoria Infirmary at Newcastle if Dr. Hume's scheme was put in force? He had been able to get the figures as to the various hospitals all over the country, and in Newcastle he found that they received from the working classes £20,000 a year. Now they wanted help and they wanted more money. The Newcastle Royal Victoria Infirmary had spent £10,700 upon insured patients last year, so that really what they had done in Newcastle, so far as the cost of in-patient relief was concerned, had resulted in a profit of something like 50 per cent. If they wiped out the voluntary system they would destroy in Newcastle alone £20,000 a year revenue and get £10,700 from the State in return. The figures represented a period which was a transition period. No doubt the cost of insured persons in that hospital and in all hospitals was likely to show an increase every year. Taking the whole country, the voluntary hospitals were already spending a sum of from £400,000 to £500,000 a year on the in-patient treatment of insured persons. That was the great fact which was interesting from the point of view that relatively the amount was smaller than anticipated. Another point in respect of National Insurance was to be remembered. Hospital beds that were formerly empty and available for accidents or special emergencies in the hospital were now filled by insured persons. Not only was the demand on those beds so great for in-patient treatment, but at the present time, on the lowest computation it could be put at, there could not be less than 10,000 patients waiting, whose cases were acute and urgent, and who needed in-patient treatment which they could not get. That in a nut-shell crystallised the position as far as Dr. Hume's paper went, and he (Sir Henry) was indebted to those hospital authorities who had enabled him to give these figures. He thought that the figures he had submitted to them spoke eloquently as showing both the injustice done to the voluntary hospitals by politicians and the relative ease with which the financial difficulty might be overcome. There were upwards of 50,000 insured persons who received in-patient treatment at the voluntary hospitals in 1913. Of course, some of these patients would have had relief and been treated as in-patients in any case because they were eligible for in-patient treatment. But there were amongst them a good many patients who had been recruited by the panel doctor. The panel doctor was really a recruiting sergeant for the hospitals. (Laughter.) It was a fact. The panel doctors were really working, and it was to their interest to do so, to put as many patients as possible in the hospital beds. There had been 50,000 insured persons so treated in 1913. That was a position created by the National Insurance Act, and that was a position that hospital managers had had to face without any sort of recognition or thanks or a farthing of money from the British Exchequer.

### The Position of Voluntary Gifts.

What was the position now as to the question of voluntary gifts? He did not think there was any material change in the attitude of the givers. It seemed to remain as it was. People, in his opinion, seemed inclined to favour voluntary hospitals more, and to stick to them more, and he believed that this was largely because of the disturbance of previous arrangements. This had made people think, and they had come to the conclusion that if the artisans were to continue their work they must have in-patient treatment, and as the voluntary hospitals had previously provided this, it was still the duty of every Christian man and woman to support its continuance. Dealing with the hospitals that Dr. Hume specially instanced in his paper, Sir Henry said he would only make this general remark—there were hospitals and hospitals, and a hospital might be prosperous to-day and five years hence it might not be prosperous. It was unfair and a very unintelligent way to conclude that the difference between prosperity and failure in a particular hospital at two given periods was dependent wholly or at all upon the attitude of the giving public towards that particular institution. He would not put it more than that. Voluntary support was not the emotional, by fits and starts, unreliable thing that Dr. Hume imagined. Voluntary support had been growing in these last twenty-five years. Sir Henry referred to the agencies that had been at work, and said that personal service had yielded extraordinary results. Since 1896 the income of the voluntary hospitals had increased from under two and a-half to over four and a-quarter millions sterling, that was by 69 per cent. Since 1909 the voluntary hospitals had laid by on an average nearly £350,000 a year. These figures could not be controverted, and he stood by them. The number of beds in the voluntary hospitals was over 50,000, or under one and a-quarter beds per 1,000 of the population.

### A System of Co-operation: A Halifax Example.

When they talked of hospital beds they must remember that as to the voluntary system the voluntary hospitals were not the only hospitals we possessed. Sir Henry urged that there should be one great system of co-operation and intercommunication as regarded our hospitals. He thought that the county was the best unit. They would have representatives on the committees just as they had representatives in connection with voluntary hospitals, who would know exactly what was going on and had to deal with the work that came before them. We had got in this country a surprising number of hospital buildings erected from the best of plans and quite up to date as hospital buildings. He was in an institution on the previous day which contained 400 beds, and where the cost of the hospital buildings he estimated at from £240 to £300 per bed. It was very difficult to find in that institution one acute case. It was used mainly for chronics, imbeciles, epileptics, and for senile and maternity cases. These cases might perfectly well be treated in a simple building costing perhaps £50 a bed at the outside, and they would then have a hospital which with very little alteration could be converted into a modern hospital and run as such. They would just want an excellent system of nursing, and the whole thing would work. There were a great many similar institutions in the country which could be used and brought into a new scheme for hospital accommodation. The taint of pauperism would be removed from those institutions. They would be then named State hospitals, and they would be made to meet the needs and necessities of the insured people of the district in which they were situate.

PATIENTS' PAYMENTS AND CLASSIFIED PAVILIONS.

What was wanted by the public was a system of classified pavilions with admission by patients' payments. He was glad that Dr. Hume had referred to this in his paper. Working-men held, and they held this very strongly, that they did not want to be under any obligation to charity, and they did not want to be under any obligation to the ratepayer, because that meant the taint of pauperism. It would be right to give these working men, by classified pavilions, the opportunity to pay. One difficulty which had stopped the development in this country of the paying pavilion had been the attitude of the medical profession. Under the system of classified pavilions which he had in view, every doctor would be paid. There would be a separate entrance to each pavilion, and if desired the patients could be attended by their own doctor. The present position generally was that the ordinary person found that the cost of nursing and treatment at home was prohibitory to the large majority of householders. They really could not meet it. This was becoming a very great difficulty, and it did not matter what the social circumstances of patients were. He maintained that each community had the hospital it deserved. This would become increasingly the fact with the pay system and with other great and much-needed reforms. The people had the matter much in their own hands. What was wanted at this moment was complete information of the hospital needs and of the total accommodation available throughout the country. By co-operation and intercommunication they could readily organise a perfected system of hospital service with the maximum of comfort and protection to the patient, and with the minimum of cost.

The voluntary hospital system was the birthright of the Englishman. It was priceless in its value to free and independent men and women. It would consequently survive the perplexities and troubles of the moment, and emerge from its troubles strengthened and extended and made equal to the requirements of the people of all classes. Not State aid but cordial co-operation and conference between municipal, local, and voluntary hospital authorities was the way out. Manchester and Leicester, to quote two notable examples, had proved this to be so. This was the direction, he was perfectly certain, in which the solution of the present problem was to be found.

Mr. J. R. THOMSON, Chairman of the Chester Royal Infirmary, said the hospital which he had the honour to represent supplied the district the population of which was very much smaller than the population to which the Newcastle infirmary ministered. The population of their city was about 40,000, and they got a number of patients from the wide area outside their district, which was not a very populous one. He should like to say, with reference to the ability and willingness of the people to contribute to their funds, that at any rate during the last eighteen months they had had the help of the people to the extent of very nearly £40,000 from that area, but it was practically entirely from those who would under no circumstances derive any benefit from the hospital. They had also received very welcome contributions on a very much smaller scale from good friends of their hospital such as the Saturday Fund, the Oddfellows, and so on. They had been busy considering reconstructing the building, and as the work was just about to be begun they hoped to have the hospital completed in six or eight months' time. A difficulty which they had to contend with was the income. He thought that their infirmary in this respect was similarly situate to a number of other infirmaries of its size and kind in the country. They had a large overdraft upon their current account, and they were working the hospital at a loss of about £2,000 a year. They were on the eve of endeavouring, with the help of the working men, to get larger contributions. It seemed to him perfectly obvious that if they were to get no help at all from the National Insurance Fund, and that if obstacles were to be placed in their way that were to be of a permanent character, such as that foreshadowed in the proposed amendment to the Truck Act, they would be placed in a very much more serious position than that in which they were now placed. He understood that the Insurance Act of this country was very nearly connected with that in Germany, where great benefits were derived from subsidies. It would be a very gratifying fact if they could be able through their Association to take steps and do their uttermost to obtain help from the National Insurance Act. The Chancellor of the Exchequer had told them that that was impossible, and yet he willingly admitted that the hospitals of this land were absolutely necessary. Why, therefore, should all other sections have his countenance and virtue and not the hospitals? The hospitals received no assistance at all under the Act. Personally he was entirely opposed to anything that would imperil the present voluntary system in operation in this country, which had been, as the last speaker, Sir Henry Burdett, had so eloquently said, the greatest blessing they had had. He hoped that something practical might be done by that Association, that they might in time secure a subsidy for the hospitals of the land.

Mr. W. STRAKER, of Newcastle, corresponding secretary of the Northumberland Miners' Association, said it might be interesting to that gathering to have a representative of the working classes taking part in that discussion, because, after all, that class must play an important part in future hospital management. He dared say that before he had spoken more than a minute or two they would discover that he was strongly biassed in favour of State provision, just as he had gathered that the last speakers were as strongly opposed to it. He was not of the Chancellor of the Exchequer's party, but he must confess that he had a greater admiration for him than Sir Henry Burdett had.

Sir HENRY BURDETT: I have every admiration for the Chancellor of the Exchequer, but not for his Insurance Act.

Mr. STRAKER: I did wonder while Sir Henry was speaking whether he was discussing Dr. Hume's paper or the Insurance Act. If we were to discuss the Act, I think it would be found that that would be one of the greatest compliments that could be paid to the present Chancellor of the Exchequer. ("Question.") Well, we shall get most out of the State Insurance not by condemning it but by seeking to improve it. Let me say this, that, after all, Chancellors of the Exchequer and Governments and even the Press will now know that they have got to do what the people want them to do.

Sir Henry Burdett: And the working men, too.

Mr. Straker: Yes. Proceeding, Mr. Straker said that a peculiarity in the criticism of Sir Henry Burdett was this, that while he was so bitterly against State provision, yet his keenest criticism of the Chancellor of the Exchequer was that he had not given them sufficient of it.

Sir Henry Burdett: No, no. My point was that he had sent the voluntary hospitals 50,000 in-patients a year and provided no money to defray the cost of their treatment.

Mr. Straker said that Sir Henry had tried to make out that the Chancellor of the Exchequer had given them money for the provision of a panel doctor to deal with simple ailments, and he thought that he might have gone further than that. He seemed to be quite prepared to accept part of the compulsory money that came out of the National Insurance scheme. If he was prepared to accept that money, what became, then, of the voluntary system? He (Mr. Straker) thought that this all showed the truth of Dr. Hume's paper that they were travelling from pure voluntaryism to the State provision of hospitals. He had pointed out that they were not going to step from one point right to the other in one jump, that there would be and that there would have to be a number of half-way houses, but, as he wisely said, nobody was likely to be content to remain there very long. The point that seemed to be missed by Sir Henry Burdett was that he did not show them how they were, by the voluntary system, to secure adequate funds necessary to meet the requirements of the people for hospital treatment. In a hospital like that in which they were assembled at Newcastle they had a waiting-list of 1,300. Many of these were bread-winners on whom others were dependent for their daily bread. How were they to meet that position? The voluntary system had failed, and failed entirely, in his opinion. (No, no.) He repeated that the voluntary system had failed to provide for those 1,300 on their waiting-list. If it had not failed, why were they waiting?

Sir Henry Burdett: Simply because the Chancellor of the Exchequer has not made proper provision.

Mr. Straker: I agree with our friend Sir Henry. It is because the Chancellor of the Exchequer has not provided sufficient funds to stop such a position of affairs existing. That is exactly my position. Mr. Straker said he wanted to pass from that and get to Dr. Hume's paper. There was one part in which he drew attention, by using a quotation from another authority, to the necessity of the working classes giving increased support to hospitals because they were the classes who received the benefits. He (Mr. Straker) on that point wanted to say this, that they were not the only classes who received the benefits, and he was sure that when he said this Dr. Hume would agree with him. They had the employing classes, who received very large benefits from the hospital treatment of their workmen, otherwise their compensation bills would be much larger than they were at present. Certainly from a business point of view it would pay that class to contribute much more largely to their hospital. They also had the general public. This was not as fully pointed out in Dr. Hume's paper as he should have liked. Every part of the public ought to subscribe to those hospitals equally. There was, however, an irony in the situation, that employers and others should object, as pointed out by Dr. Hume, to contribute to voluntary hospitals such as this because of State legislation. But there was a cause for that State legislation, and he begged to submit that the cause of it was the need of it, and the need of it was created because they did not adequately subscribe beforehand. By withholding their subscriptions now the irony came in that they were only making absolutely necessary more of that State legislation which they were objecting to. The next point he wanted to deal with in the paper was with regard to payment from patients. Their system in Newcastle when they took voluntary pay from patients was the same as what was done elsewhere, but he thought what was meant in Dr. Hume's paper was that there should be a method of compulsory payment for services rendered.

Dr. Hume: No, no.

Mr. Straker: I am glad of that. Then the suggestion of that being one of the remedies altogether falls to the ground because we have it now, and if it is not to be something more than we have, then it will not make any greater provision for us than we have at the present time. Mr. Straker added that he wanted to get to the last point where Dr. Hume dealt with State provision. He stated that one of the evils which he feared with the class that would most benefit at the hospitals if they were made free were the very class that would not be called upon to pay towards them. He submitted respectfully to Dr. Hume that that was the old fallacy that the direct ratepayer was the only man who paid, forgetting that after all every man who paid rates was forced to get this out of the people he employed. After all, the worker would pay his share, and probably the larger share, towards a State-provided hospital.

### Working-men and Inquiries.

If anything would place the stigma of pauperism upon a man it would be an inquiry into his circumstances before he could receive assistance. Well, Dr. Hume did not propose that, but it would be very objectionable to have any inquiry made whatever into people's circumstances before receiving treatment. They had an enormous number of people on their waiting-lists, and they had that in connection with their hospital long before there was any State Insurance Act. At any rate they had that in Newcastle, and he could speak of those. They had had some even dying before their turn came, and widows and orphans left because of the inadequacy of their hospital provision. Had the time then not come when they should regard hospital treatment as one of the duties of the State to make provision for, and that it should be a necessary equipment of that fully co-ordinated family-like life which ought to exist? It had been suggested that there was a danger to the State of such a provision becoming bureaucratic. He did not see any need for it or any danger of that. In education and in much other work which came under the laws of the country the State deputed that work to municipal and other councils and other authorities, and they might be sure that it would be on similar lines by which State hospitals could be managed.

### The Voluntary Work of Paid Officers and Committees.

Mr. Howard J. Collins, House Governor, General Hospital, Birmingham, said that the paper of Dr. Hume must be very valuable to all officials and workers in hospitals. All of them knew it was not every member of a staff who would be willing to look on such a side of the picture as Dr. Hume. An important point raised in the paper was the fact that the expenditure on their hospitals by a natural process had grown so enormously that the giving by the general public had not been adequate. He quite agreed with Mr. Carnt in his view that they wanted a little more energy on their part, and still more assistance from their friends, to overcome the present difficulty. There had been an enormous increase in surgical work in hospitals, and there had been considerable expense involved in respect of surgical appliances. In his thirty years' experience the increase was eight- or nine-fold. The question of the cost of education was a very thorny one, and one that was exercising the minds of a good many people, and especially that part as to how far hospitals provided education for medical students. There was more than the annual subscription list about a hospital. Reference had been made to payment by patients. Payments by patients were voluntary gifts as much as any annual subscription. These gifts helped others who needed some help, and he believed that in that lay the true spirit of voluntary work. He was prepared to fight for voluntary effort, and when he spoke of this they all knew the amount of voluntary work done by the physician and surgeon. This work, he thought, was well recognised, but he did not think that the voluntary work of the committees or that of the paid official was sufficiently recognised. He was quite sure the amount of expenditure was kept down by them, while the salaries, especially of the nursing staffs, were a burning question. In many ways they had evidence of the work carried out at the hospitals being purely of a voluntary character, and when it was said that the voluntary system was on its trial he certainly pleaded "Not guilty."

Alderman Sir Riley Lord, of Newcastle, said he agreed with Dr. Hume, and was "confident that the voluntary system will fail to meet with anything like adequacy the approaching demand for increased hospital accommodation." In Newcastle they were faced with a position which had been already indicated by Mr. Straker. They had a waiting-list of about 1,300 persons. They wanted about £100,000 to enlarge their hospital, but the greatest difficulty would be the maintenance subsequently, which would mean anything from £8,000 to £10,000 a year larger income. He confessed that although he had been chairman of the subscriptions committee for nine years he did not quite see where the £8,000 or £10,000 per year was going to come from. He was entirely on the side of the voluntary system of hospitals, but he thought they were entitled to some State assistance, and that would mean necessarily some State control. He did not think that the Insurance Act had appreciably increased the number of patients or appreciably increased their expenses.

Business Arrangements in the Subscription Department.

Sir Henry Burdett: It has increased your waiting-list.

Sir Riley Lord: The waiting-list is increased, but perhaps it would have increased even if the Insurance Act had not been passed. The reason there had not been a considerable or appreciable increase was because their hospital was always full, and they could not put any more in. It did not matter what Insurance Acts were passed, and meant the sending in of more patients; they could not take them in because the place was always full. He thought that the Government should come to their aid by providing money to enlarge their hospitals and also maintain them subsequently. But this should be done in proportion to the needs of a hospital and in proportion to the voluntary assistance given to support that hospital. He was decidedly opposed to Mr. Straker's proposition that the hospital should be placed entirely in the hands of the State. He would say to their friends connected with other hospitals that it was necessary to have business arrangements introduced into the subscription department. In Newcastle they rearranged their subscription department in the year 1895, and they had more than doubled the income in that department by the arrangements which they then made. But they could not face the effort to build a large enough hospital without some assistance from the Government, and he hoped that something of the sort would come about. With regard to patients' payment, Dr. Hume had referred to Manchester, where there was an income of £4,000 a year from patients. They could not introduce a system like that very well in Newcastle, because all the working-men did already subscribe, practically speaking. They subscribed weekly, and they had the amounts deducted from their wages, or they subscribed to the Hospital Sunday Fund, or in various ways, and they could not very well ask them to come to the infirmary, and when they were there to become paying patients as well. He had addressed scores of meetings throughout Northumberland and Durham. He was always welcomed, and he never once had a refusal. They had always three resolutions put at their meetings. The first was: Do you approve of subscribing to the Newcastle Infirmary? The second was: How much will you subscribe, one penny a week? The third was: Will you have it stopped at the offices where the wages are paid so that we may get the money regularly? That was really the source of their income. Their backbone was the weekly contributions of the workmen. They began from this source in 1895 with £4,000 for the year as the workmen's contributions, and now they were up to £21,000 or £22,000 a year.

Renewing Withdrawn Subscriptions.

Dr. D. J. Mackintosh said that in Glasgow during the first year of the working of the Insurance Act general and employees' subscriptions to the amount of £123 were refused on account of the Act. This, however, was more than made up by new subscriptions received and by increases in the amount of employees' subscriptions owing chiefly to better times in the shipyards. He did not think they need fear anything in respect of the voluntary system.

The Earlier Sunday Fund?

Dr. Morgan, Sunderland, said that one of the greatest misfortunes that could happen to England was that they should have to give up the voluntary system and rely upon State aid. It had been very happily said by one of the speakers during the discussion that their hospitals contributed to many who passed through as patients. That was perfectly true, and if those hospitals did not exist the general public would be the sufferers to a large extent. He thought that there should be a committee of inquiry in every case of admission to a hospital so that improper cases did not impose on them. He thought there should be an opportunity given to some patients to contribute something which would relieve in a great measure the burdens of maintenance in many hospitals. In Sunderland they were before their friends in Newcastle in respect of the Hospital Sunday Fund, he thought they were in advance of them by four years. The Lord Mayor of London had said that Sunderland had set an example to the whole country by organising workmen's subscriptions which they in Sunderland and Newcastle to-day derived so much benefit from.

